# Predation risk is a function of seasonality rather than habitat complexity in a tropical semiarid forest

**DOI:** 10.1038/s41598-021-96216-8

**Published:** 2021-08-17

**Authors:** Anthony Santana Ferreira, Renato Gomes Faria

**Affiliations:** 1 Programa de Pós-Graduação Em Ecologia E Conservação, Universidade Federal de Sergipe - UFS, São Cristóvão, Sergipe 49100-000 Brazil; 2 Programa de Capacitação Institucional, Instituto Nacional de Pesquisas da Amazônia - INPA, Manaus, Amazonas 69067-375 Brazil; 3 Departamento de Biologia, Universidade Federal de Sergipe - UFS, São Cristóvão, Sergipe 49100-000 Brazil

**Keywords:** Behavioural ecology, Ecological networks, Behavioural ecology, Ecology, Ecology, Environmental sciences

## Abstract

Predator–prey dynamics are some of the most important species’ interactions in the natural structuring of communities, and are among the more complex ecological processes studied by ecologists. We measured predation risk using artificial lizard replicas to test two competing hypotheses regarding predation pressure in semi-arid environments: (1) predation risk is dependent on the habitat structural complexity; and (2) predation risk is dependent on seasonality. We placed 960 lizard replicas along three sites with different physical structures and in both dry and rainy seasons for seven consecutive days in a caatinga area in northeastern Brazil at Grota do Angico Natural Monument (GANM). Birds were responsible for the majority of attacks and more frequently on artificial lizards placed in trees. Attacks focused on the most vulnerable areas of the body (head and torso), proving that were perceived by predators as true prey items. We found that predation risk is not dependent on the habitat structural complexity, but rather dependent on the caatinga seasonality, with the overall attack rate being 19% higher in the dry season. Our study suggests that potential predation risk is highly context-dependent and that seasonality consistently drives of trophic interactions strength in the caatinga, an important ecological finding that could contribute to better understanding the complex evolution of predator–prey interactions within communities of animals living in different habitats.

## Introduction

Predation is one of the most important processes in the functioning of ecosystems, with predators acting as key regulators of prey populations^1–3^. This selective pressure can determine demographic parameters, geographic distribution, population structure, evolution of morphological characters, life history traits and behavioral strategies, of both prey and predators alike^4–7^. The impact of predation on prey populations is best estimated using attack rates^8–10^, but these are notoriously difficult to measure in natural systems because of the rarity of observing predation events and fast consumption or removal of prey carcasses by predators^2,11,12^.

Multiple factors can affect predation risk, including habitat structural complexity and seasonality. Concerning predation avoidance, prey often respond to predation risk by increasing refuge use^19,25,36,37^. There is also large consensus that habitats with high structural complexity are expected to offer a large diversity of microhabitats that can be used as refuges and used by prey organisms such as lizards for cryptic concealment, thereby reducing predator foraging impacts^11,38–40^. Further, caatinga vegetation changes dramatically over the year due to its very marked seasonality and hence is expected to have important biological and ecological consequences on predation pressure. Consequently, animals should adjust their refuge use in ways that reflect increased predation risk^37,41,42^. If the extent of predation risk is not measured taking into account seasonal variations, especially in environments strongly marked by them, such as caatinga biome, it is not possible to distinguish which of these factors is more important in driving predation risk. However, the role of predation avoidance in the process of refuge use with habitat structural complexity as a function of seasonality in the caatinga biome remains unstudied.

Alternatively, an approach used in evolutionary, ecological and ethological studies consists of recording predatory attacks on soft replicas to quantify such events in nature, allowing the comparison of predation rates observed in different environments under different conditions^12–15^. This technique is particularly useful because it allows identification of potential predators via examination of imprints on the replica surface^16–19^, being effective to test different ecological hypothesis in field experiments (e.g.,^20–22^). The use of this simple and inexpensive technique has provided accurate and reliable estimates of potential predation risk, with highly illuminating results in studies involving bird nests^23,24^, lizards^2,11,25–29^, amphibians^15,19,30–32^, snakes^33–35^, and also small mammals^14^.

To better understand this issue, we used attack rates on soft replicas as surrogates for lizards (sentinel prey) to directly quantify predation risk and examine the relative role of habitat structural complexity and inter-seasonality variation in determining predation risk in three different habitats in the Grota do Angico Natural Monument (GANM) in the Caatinga of northwest Sergipe, Brazil. The primary goal was to empirically test two hypotheses regarding predation risk: (1) is predation risk dependent on the habitat structural complexity, i.e., does predation risk increase in less complex habitats; or (2) is predation risk dependent on seasonality, i.e., does predation risk increase in the dry season due the seasonally deciduous nature of caatinga vegetation that results in a loss of protective cover for potential prey? We further investigated predator attack patterns with respect to potential predators (birds, giant ant, lizards and mammals) and the location of attacks on a lizard’s body. Here, we provide empirical evidence that predation by visual predators such as birds is strongly modulated by local abiotic conditions in a lizard’s community in a poorly-studied Brazilian biome highly threatened by anthropic actions.

## Results

### Environmental structure

**Figure 1:**
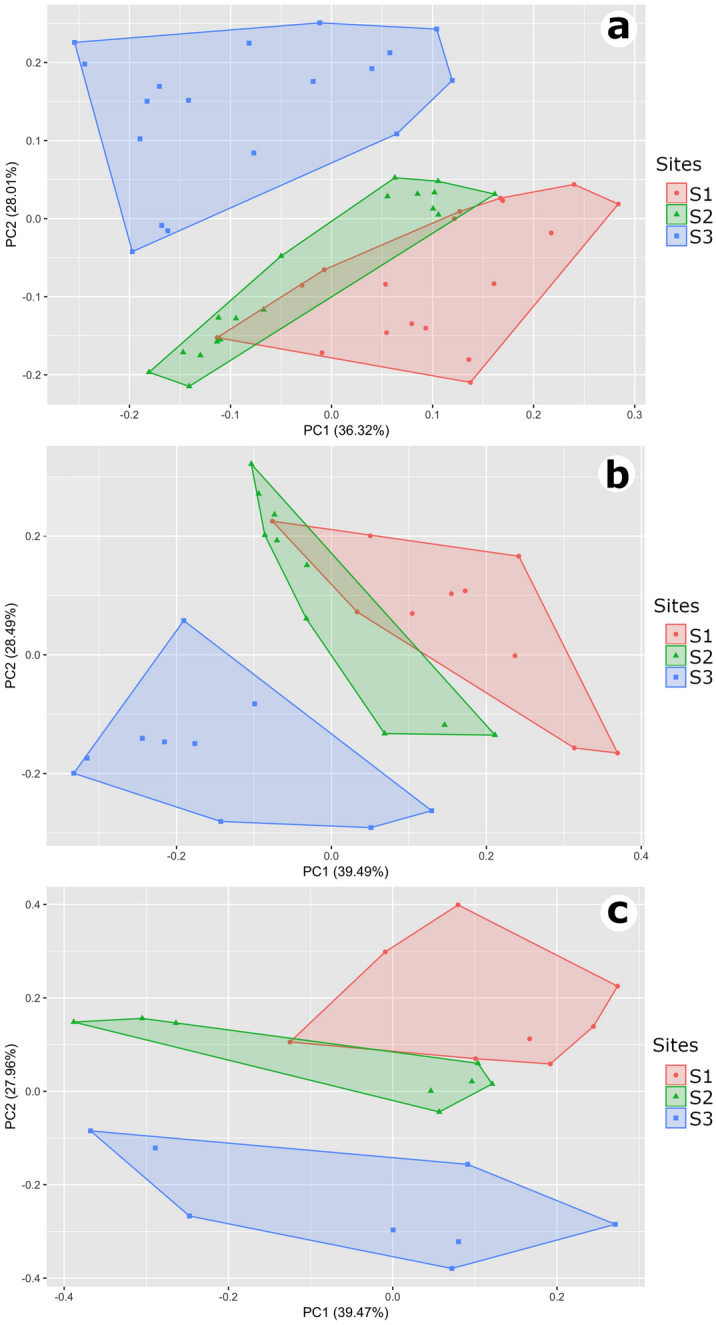
Multivariate environmental space at GANM. Principal Component Analysis for the eight environmental variables measured at each sampling site (S1, S2 and S3) for 18 consecutive months. (**a**) General structure (all 18 months). (**b**) Dry season only. (**c**) Rainy season only.

The environmental variables measured for each site over 18 consecutive months are shown in Table 1. The experimental sites differed considerably in relation to environmental variables (Table 1; Fig. 1). Tree, number of stems, rocks and bromeliads were higher at S2. Tree diameter, leaf litter and fallen trunks were higher at S1. Vegetation was especially sparse and low at S3.

**Table 1:** Environmental variables estimated at three study sites at Grota do Angico Natural Monument (GANM), Sergipe, Brazil. Values correspond to the mean ± SD of data collected for 32 quadrat frames in each site (spread along a 5-km-long transect with a minimum distance of 2 km between them).

Environmental variables	Sites		
	S1	S2	S3
	$\overline{x}\pm sd$	$\overline{x} \pm sd$	$\overline{x}\pm sd$
Exposed soil	0.69 ± 1.67	2.25 ± 4.01	11.7 ± 6.93
Leaf litter	23.15 ± 2.05	19.63 ± 5.05	12.8 ± 6.92
Rock	1.25 ± 1.09	3.02 ± 2.58	0.04 ± 0.17
Number of stems	1.39 ± 0.52	2.46 ± 1.12	1.52 ± 0.78
Distance of nearest tree	19.06 ± 12.28	12.62 ± 11.38	15.01 ± 9.47
DBH of the nearest tree	18.39 ± 8.34	16.27 ± 6.83	10.89 ± 5.36
Number of fallen trunks	1.04 ± 0.83	0.98 ± 0.81	0.79 ± 0.86
Bromeliads	0.06 ± 0.18	3.50 ± 4.65	–

*DBH* diameter at breast height of nearest tree. For information on the three sites see “Methods”.

The first axis (PC1) of the PCA for general structure explained 36.3% of the variation in the environmental variables data, whereas PC2 explained 28%. The first axis (PC1) of the PCA for dry season summarized 39.5%, whereas PC2 summarized 28.5%. The first axis (PC1) of the rainy season PCA explained 39.5%, whereas PC2 explained 28%. The values for PC1 and PC2 were similar among the three PCA analyses, however, the environmental variables related to the variation of these axes were different.

The PC1 was related to the greatest number of stems and rock for general structure and dry season, while for the rainy season this involved the greatest number of stems and number of fallen trunks. Also, the PC1 was inversely related to the distance to nearest tree and nearest tree DBH for general structure and rainy season, and to the distance to nearest tree and proportion of exposed soil for dry season. The PC2 was related to the higher proportion of exposed soil and number of fallen trunks for general structure, DBH of the nearest tree, and leaf litter for dry season, and to the greatest number of rock and bromeliads for rainy season. The PC2 were inversely related to the higher proportion of leaf litter and DBH of the nearest tree for general structure, to the greatest number of fallen trunks and exposed soil for dry season, and to the higher proportion of exposed soil and number of stems for rainy season (See Supplementary Tables S1, S2 and S3 for values of all axes).

Differences in the environmental structure among the three experimental sites were observed for all 18 months (Pillai trace = 0.912, *df* = 102, *P* < 0.001), in the dry season (Pillai trace = 0.952, *df* = 54, *P* < 0.001) and in the rainy season (Pillai trace = 0.936, *df* = 42, *P* < 0.001). The measured environmental variables did not overlap (except for a slight overlap of the S1 and S2 in the general structure and in dry season) in multivariate environmental space (Fig. 1a,b,c).

### Predation assessment

Nine hundred and forty out of 960 (98%) lizard replicas were recovered from the experimental sites, of which 130 (14%) showed some sign of predation (Supplementary Table S4). Six replicas were lost in the rainy season and 14 in the dry season. There was no significant difference in attack rate between experimental sites (intercept ± SE = -2.11 ± 0.56, Wald test, *P* = 0.07; experimental sites (S1) ± SE = -0.22 ± 0.25, Wald test, *P* = 0.37; (S3) ± SE = -0.07 ± 0.24, Wald test, *P* = 0.78), with an attack rate of 4.6% at S1, 4.9% at S2 and 4.1% at S3 (Supplementary Table S4). However, we observed a significant effect of seasonality on attack rates (intercept ± SE =  − 1.45 ± 0.11, Wald test, *P* < 0.001; season ± SE = -1.52 ± 0.24, Wald test, *P* < 0.001), with replicas in the rainy season experienced an attack rate of 0.08, while the predation rate in the dry season was about three times greater. In the rainy season, the highest predation frequency occurred at the site with the highest structurally complex vegetation (S2 with 4%) followed by sites S1 and S3, with 3% and 1%, respectively. During the dry season, the highest frequency was in the less structured site (S3 with 7%), followed by sites S1 and S2, both with 6% (Supplementary Table S4).

**Figure 2:**
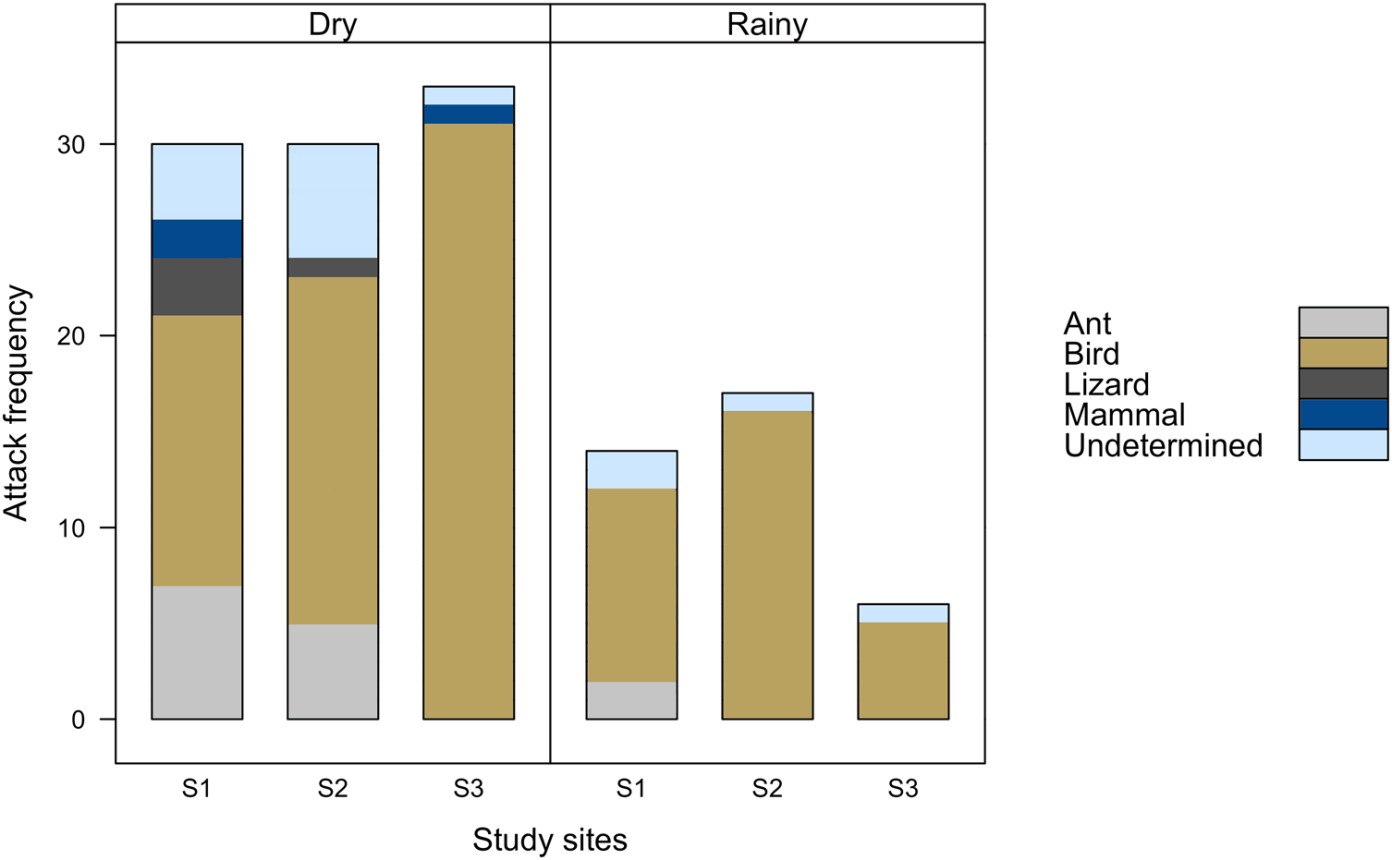
Frequency of attack by the different categories of predators on the artificial lizard replicas arranged by season (dry and rainy) for the three sample sites. For number of lizard replicas used in each experimental site see Table S4.

The use of microhabitats varied among experimental sites depending on their local availability, with 66% placed on the soil, 24% on rocks, and 10% on trees. Although with fewer placed replicas, the trees showed a higher predation rate with 47% attacks, followed by soil and rock with 11% and 6%, respectively (Supplementary Table S4). The attacks on 94, 14, 4 and 3 models could be attributed to birds, ants, lizards and mammals, respectively, while the predators responsible for the attacks on 15 models remained unidentified (Fig. 2). Our attack rates on replica lizards likely represent a conservative estimate because all replicas that were attacked by predator classes of under-five attacks (lizards and mammals; n = 4 and 3, respectively), giant ant attacks (n = 14), or were missing upon collection (n = 20) were removed from analysis because we established a cut-off value below five, ants are not usually considered as lizard predators and the fate of the absent replicas could not be determined. Therefore, we only considered the attacks of birds, which were responsible for a majority of overall replica lizard attacks (72%).

The body regions of the replicas were attacked similarly in the three experimental sites (*χ2* = 2.08, *df* = 4, *P* = 0.72). However, the attack position on lizard's body differed significantly in the number of attacks in comparison to expectation (χ2 = 234.93, *df* = 2, *P* < 0.001), suggesting that the predators attacked the models non-randomly. In particular, the head was attacked significantly more (114 attacks; 59%) than expected by chance (225.04 expected attacks; χ2 = 36.367, *df* = 1, *P* < 0.001). The torso received 34% attacks and lastly tail with 7%. There were no records of replicas with attacks on limbs.

## Discussion

Our results showed that predation risk on lizards was not directly related to structural differences between habitats. This suggests that they were perceived as a continuous habitat, at least by birds, which were the main predators, therefore, lending no support to the hypothesis that habitat structural complexity drives predation risk. Instead, we found a significant relationship between seasons and predation risk, with a noticeable higher predation rates during the dry season, lending strong support to the alternative seasonality hypothesis. Our study constitutes one of the few empirical studies that identifies potential mechanisms underlying predation risk in a semi-arid environment, where predation rates were more influenced by local abiotic conditions, providing more evidence to compose the scenario of mixed results between different regions, as suggested by some studies (e.g.,^10,21,43–45^). In addition, birds selectively attacked the replicas' head and more often on replicas placed in trees than on the most available microhabitats with them (soil and rock), indicating that they perceived lizard replicas as real prey items and were aimed to attack the most vital body region of their target organism in its main foraging microhabitat. This outcome was expected based on behavior of predators observed in other studies^14,18,19,22,46–48^.

**Figure 3:**
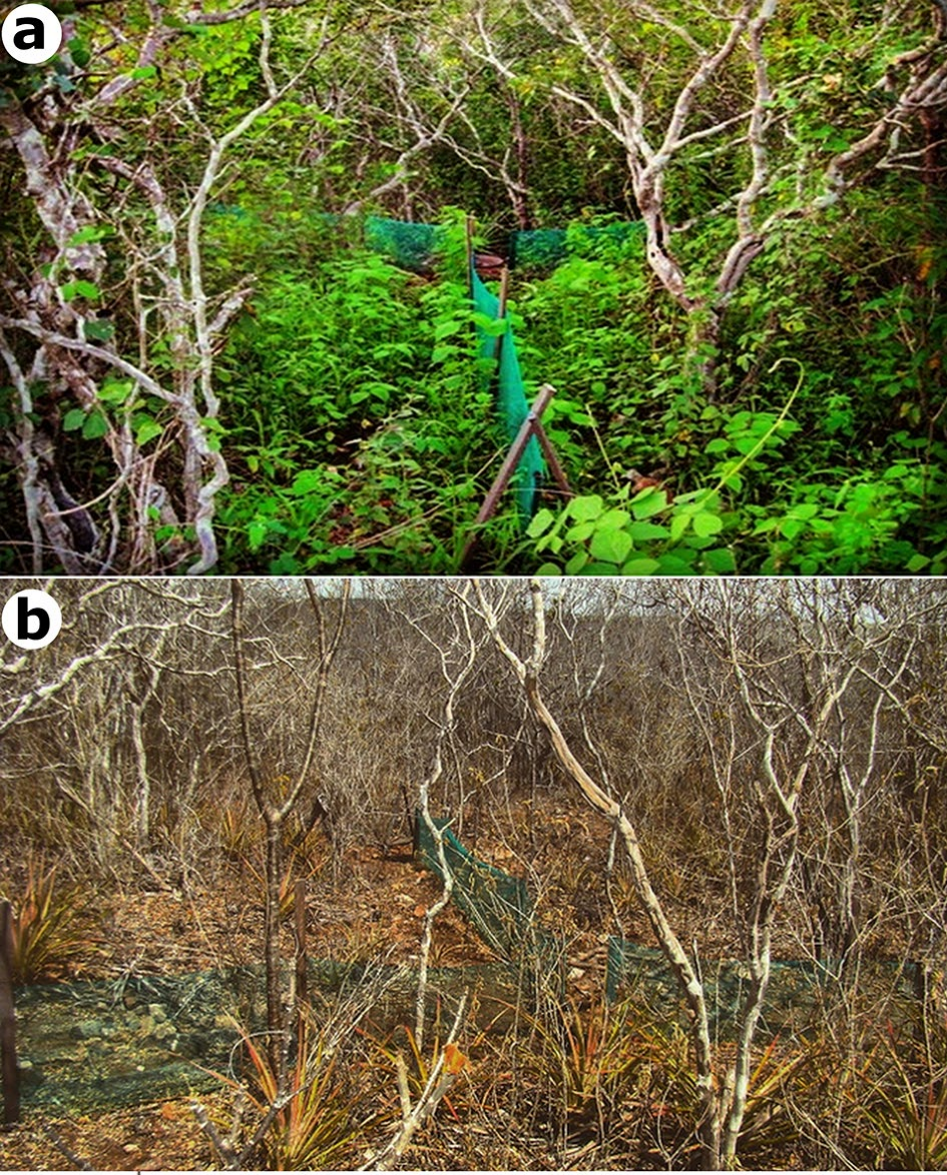
Overall characterization of landscape change due to seasonality at the Grota do Angico Natural Monument–GANM, Sergipe, Brazil. (**a**) Experimental site during the rainy season and (**b**) Same experimental site during the dry season. All photographs taken by Anthony Ferreira.

The climate and vegetation of the caatinga are very heterogeneous, which may respond for marked differences in local biological communities^49,50^. We observed a non-significant trend of increase in the observed number of attack at the less-structurally complex site (S3) during the dry season, but this site is located near the banks of the São Francisco River. A possible explanation is that during the dry season, caatinga vegetation loses its leaves, becoming completely xeric (Fig. 3), which drastically reduces humidity and overall resources availability (e.g., refuge and food items). Under such circumstances, animals incessantly seek for places with water for hydration, to the detriment of their safety. As site S3 is located on the river bank, this likely caused an increase in predator abundance during the period of water scarcity, which is generally associated with higher predation pressure^39,51,52^. Similarly, other studies have found a strong seasonal shift in the attack rates on lizards, with greater attacks frequency during the dry season^21,53^.

In contrast, the vegetation flourishes in the rainy season (Fig. 3), increasing the availability of resources^54,55^, and more structurally-complex environments become more attractive for prey like lizards^39,56–58^. We also detected a non-significant trend of increase in the observed number of attack at the more structurally complex and denser sites (S2) during the rainy season. Castilla & Labra (1998) found a negative, marginally significant rank correlation between predation rates on replicas with lizard density in both seasons, indicating that lizards tended to be more abundant in areas with lower on-replica predation intensities. Some studies have reported increased predation risk for lizards related to the abundance and diversity of predator^39^, lizard density^59^, and the abundance of alternative prey^21^. However, the increase in these factors does not necessarily mean they will contribute to increased predation (see^21,60,61^). Regrettably, in our study, the relationship between predator abundance and density, levels of food availability (alternative prey) and lizard density with predation risk were not investigated. Further research is needed to accurately examine the contribution of these factors to the predation risk, especially in this type of environment where food availability fluctuates seasonally.

A variety of animal species are considered as lizard predators, and predation of static replicas is attributed, mainly, to visually-oriented predators that forage actively^11,62^. In the current study, predatory birds were responsible for a majority of attacks on artificial lizards (72%), a fact which fits with the classification of birds as the main predator of small herpetofauna in many ecosystems (see^2,11,26,53,63–65^). Birds are excellent and voracious predators, precisely because their high metabolic rates result in high food demands^7^. Their excellent visual acuity allows them to perceive and attack prey even when immobile^66^. We suspect that majority of the bird attacks on artificial lizards came from diurnal predatory birds abundant at our sites. Ruiz-Esparza et al. (2011)^67^ carried out a daytime and nighttime inventory of the avian fauna of the Grota do Angico Natural Monument (GANM) and revealed the presence of at least 140 species, where majority of the predatory birds are diurnal or crepuscular and forage on trees or near the ground. This trend was similar to other semi-arid scrublands across Brazilian northeast (e.g.,^68–70^). However, we did not distinguish between daytime and nighttime attacks. A study that did this found no significant difference in the attack rates on artificial lizards from birds during the day or night^21^. They found that diurnal birds were the main contributors to the predation, especially at dawn and dusk, when many birds were foraging and lizards thermoregulating.

Although it is generally assumed that ants are not considered frequent predators of lizards, we found an interesting frequency of replicas attacked by the giant ant *Dinoponera quadriceps* (11% of incidents). Despite the potential impact of carnivorous ants on lizard populations, data on the actual intensity of predation is rare and fragmentary, often based on anecdotal and casual observations (e.g.,^71–73^). Furthermore, it is probable that predation events by invertebrates on vertebrate assemblages are more frequent in nature than has been documented^74–77^, and large predatory invertebrates may be important predators of small lizards, even though invertebrates are often overlooked in this capacity^78^. Therefore, the assumption that carnivorous ants do not prey on vertebrates with significant frequency, especially smaller or juvenile lizards should be validated on a case by case basis. However, due to skepticism surrounding this type of predation, we excluded ants from data analysis, but we warn that exclusions like this may produce spurious results. For example, it may inflate the relative frequency of other predatory animals or reduce the overall predation rate (see a similar case for attacks on artificial lizards from invertebrate predators—huntsman spiders and centipedes^21^ and daytime and nighttime predation by mammals^22^). The exclusion of attacks by the giant ant, in our case, did not change the overall pattern of results (see results with ants in Supplementary Table S5).

The use of artificial replicas is very advantageous because it allows testing rare events that are difficult to observe in nature or expensive to perform in the lab with experimental animal^12^. In addition, it allows researchers to display a large number of these replicas in the field, usually in a simple and inexpensive way, permitting a high statistical power to test several ecological hypotheses in field experiments^14^. However, this technique has obvious and important drawbacks: the absence of motion and typical prey odours, spectral colour differences in comparison to the living animals and usually are displayed continuous in the field without distinguishing between daytime and nighttime attacks. All these issues may reduce the reliability of results if they are not taken into account (see^12,14,22,31,79^). Nevertheless, as a similar experiment design was used in all experimental sites, the observed differences in attack rates can be considered as estimates of relative differences in potential predation rates. And taking into account the limitations of the technique and our experimental design, our results can only be considered as viable estimates of the potential predation rates on immobile lizards that did no detect the predator’s approach, small lizards or in juvenile stages and for diurnal birds as predators.

The dearth of ecological and behavioral studies in semi-arid areas and the peculiarities of these environments raise interesting questions regarding the assemblage dynamics and community processes. Results presented here using a simple and low-cost experiments confirm the strong association of predation risks with local abiotic conditions caused by seasonal changes rather than structural complexity of habitats in a semi-arid caatinga of northeastern Brazil. Thus, our study, suggest consistent guidelines of the relative importance of these processes in determining predation risk in the caatinga domain. To our knowledge, this is the first study done in the domain of the caatinga that provide empirical evidence of the complex process of predation pressure using an experiment based on lizard replicas. It is hoped the current study will stimulate further standardized comparisons of trophic interactions on predation risk in the real world, contributing to a better understanding of these key biological interactions, and thus illuminating our search for their drivers and consequences.

## Methods

### Study area

This study was conducted in the Grota do Angico Natural Monument—GANM (09° 39′ S; 37° 40′ W), a 2,183 ha protected area (mean elevation 100 m a.s.l.), located on the right margin of the São Francisco River, Sergipe state, northwest Brazil (Supplementary Fig. S1). The study area lies within the seasonally dry tropical forest in the semi-arid region of Brazil (Fig. 3), known as caatinga which covers approximately 850,000 Km^2^ (some 10% of Brazil’s land surface), and comprises a large part of northeastern Brazil^80^. The caatinga is also the most impacted and the least studied of all Brazilian biomes, being subjected to great anthropic pressure due, primarily, to agriculture expansion and extensive livestock farming and wood extraction^81^. This has resulted in a 45.6% reduction of the original vegetation cover^82^. The predominant phytophysiognomy at GANM is classified as deciduous and composed of semi-arid thorny woodland and xerophytic formations growing on shallow and rocky soils^83^. The arboreal-shrub vegetation at GANM is secondary successional, with a sparse understory and herb layers^83^. The regional climate is arid (BShw—classification of Köppen), with high solar radiation (with an accentuated diurnal range, but with means of around 30 °C during the dry season), low cloud cover, low relative humidity, and rainfall that is both annually low (annual average around 500 mm) and irregular within and between years^84–86^.

### Experimental sites

At the GANM, eight standardized sampling stations were maintained along three sites with different physical structures. For the present study, we performed replica-based predation experiments in 24 stations in these three research sites which are spread along a 5-km-long transect (with a minimum distance of 2 km between them). The general features of these experimental sites are: S1: an area with arboreal-shrubby vegetation, with shallow, clayey, stony soil covered by leaf litter with few apparent rock outcrops, and few bromeliad patches. S2: an area of sparse shrub, on shallow, stony, clayey soil, and with extensive patches of bromeliads scattered throughout the entire area. S3: an alluvial terrace with sandy soil and predominantly herbaceous-shrub vegetation, dominated by *Croton campestris* (locally known as *velame do campo*), and scattered large trees.

To test for effects of habitat complexity and seasonality on predation risk, we characterized the physical structure of each station and site in monthly visits of three consecutive days from January 2012 to June 2013; and to determine the impact of seasonality we used operational definitions based on historical averages for precipitation for the area between 2003 and 2013 (source: SEMARH/SE). Months where precipitation exceeded 45 mm (historical average) were considered part of the rainy season. Values lower than this threshold were assigned to the dry season. Accordingly, the rainy season is from April to August, and the dry season from September to March (Supplementary Fig. S2).

To quantify vegetation complexity, we used a square frame made of 0.5 × 0.5 m PVC tube (42.16 mm), divided into 25 smaller and equal squares (0.1 × 0.1 m each). From the central point at each station, we threw the frame four times in each cardinal direction (N, S, E and W), totaling 32 times per site. At each quadrat frame, the following parameters were estimated: percentage area with leaf litter, rock and exposed soil. These types of coverage were assigned to each sub-Sect. (0.1 × 0.1 m) of the frame when a given coverage type had > 50% for that sub-section. Then, these data were converted to an overall cover percentage for the whole quadrat frame. We also measured the number of stems (from the central point of the quadrat frame, a 1 m-long pole was rotated 360º at 25 cm above the soil surface, and the number of stems that it touched were counted); distance and trunk diameter of the nearest tree (from the central point of the quadrat frame, and including only arborescent vegetation with diameter at breast height (DBH) ≥ 5 cm); number of fallen logs (within quadrat frame and with DBH ≥ 10 cm and length longer than 1 m); and area covered by bromeliads within quadrat frame.

### Lizard replica construction

**Figure 4:**
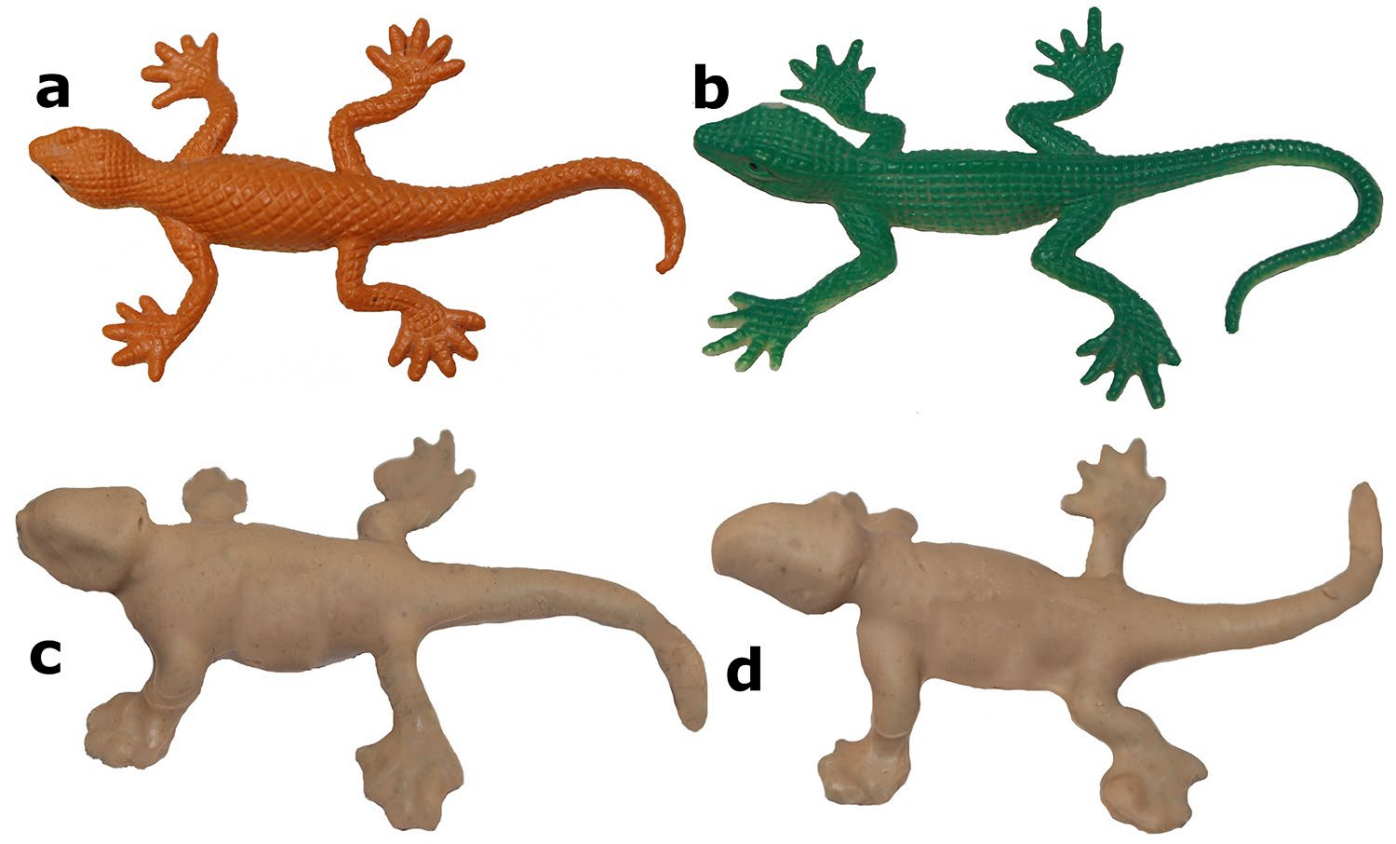
Artificial lizard replicas (8 x 75 mm) used in the field predation experiments. (**a**, **b**) Original replicas made of flexible plastic. (**c**, **d**) Replicas manually covered with brown-colored non-toxic modelling clay (plastiline). All photographs taken by Anthony Ferreira.

We used commercially available flexible plastic replica lizards which we coated with non-toxic and odourless modelling clay. We dipped lizard replicas into melted Plastalina modeling clay allowed some of the liquefied clay to run off, and then let the clay on the replicas cool. The clay formed a thin layer without modifying the body form of the replicas, while providing a soft layer retains predator marks^17–19^. We used clay replicas as surrogates for a lizard community. Eleven species of lizards have been documented from the GANM region with morphological differences in size, color, shape and activity periods^87,88^. All replicas were standardized in size (8 mm wide, 75 mm long), color (brown) and shape (Fig. 4). Although the replicas are not similar for all adult lizard species present in the study area (e.g., *Ameiva ameiva*, *Ameivula ocellifera*, *Tropidurus hispidus* and *T. semitaeniatus*), we used standard lizard replicas as a model to address this issue and also to not introduce extra variables into the analyses. There are no species in the GANM that at the adult stage are smaller, and thus, outside the range of potential predators of the 75 mm long clay replicas. However, using brown-colored and odourless replicas may have some limitations (see^11,12^). On the other hand, Castilla et al. (1999) showed that replicas experienced high attack rates regardless of the contrast between body color. Nevertheless, brown color was considered the best option, both because it represents the most common dorsal color pattern among the lizard species of the region, and because it does not cause great contrasts with the microhabitats where they were placed (soil, rock and trees). Furthermore, attempts to emulate color are far more likely to create problems than provide solutions (see also^11,38,89^). The proportions of the head, torso and tail were measured with the aid of a digital caliper (accuracy 0.01 mm) and were 1.16, 2.26 and 4.08, respectively.

### Experimental design

Replica-based experiments were carried out on seven consecutive days (period in which lizards are exposed to their potential predators, being active or even sleeping), and in two seasons (July—rainy season and November—dry season) during 2013. At each station, we positioned 20 lizard replicas (about 3–5 m spacing), a total of 160 replicas per site (480 replicas per season and 960 in total). All replicas at each experimental site and in both seasons were put in place in a single day. They were positioned to ensure they were all equally conspicuous being placed on surfaces where basking lizards had been observed previously. Replicas were located in places visible from overhead, and in a position that simulated an immobile basking lizard. Replicas were placed in the same orientation (horizontally placed), in three different microhabitats: soil, rock and tree (at a height of 1–2 m above the ground, and fixed to the trunk with a drop of odourless acrylic glue to prevent being blown away by the wind). These microhabitats were typical of those used by all the lizards native to the study area (e.g., *Acratosaura, Ameiva, Ameivula*, *Brasiliscincus*, *Tropidurus*, *Vanzosaura*). Inter-site variations meant that the number of replicas in the three microhabitats at each station varied between sites, this lack of standardization is natural, as they represent the microhabitats available to the real lizards in each site, and therefore, reflects natural variability in prey species composition.

**Figure 5:**
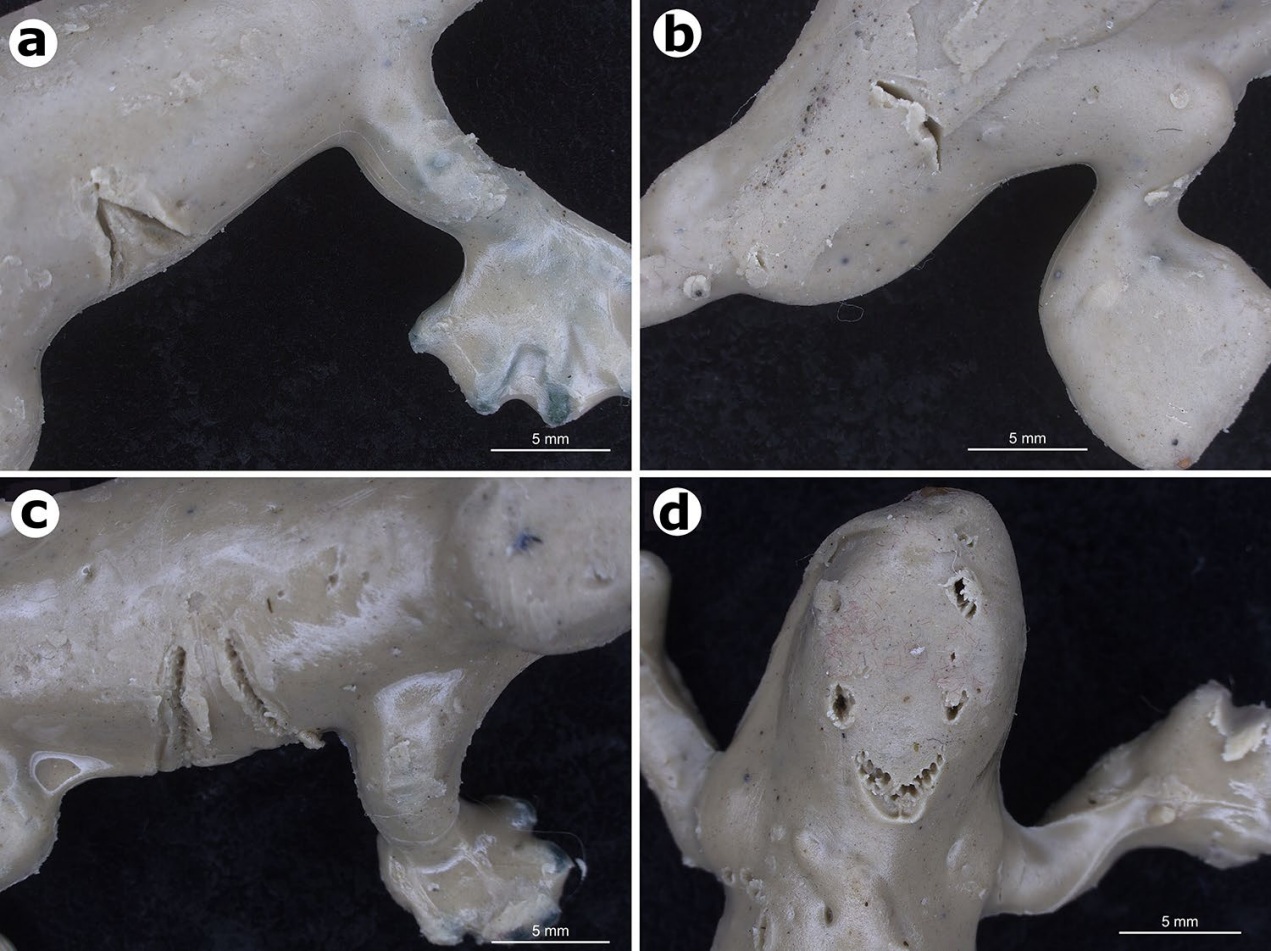
Predator bite marks on lizard replicas coated with modelling clay (plastiline) from field predation experiments. (**a**) Bird mark. (**b**) Mark from giant ant *D. quadriceps*. (**c**) Lizard mark. (**d**) Mark by an omnivorous/carnivorous mammal. All photographs taken by Anthony Ferreira.

Subsequently, all replicas were inspected once a day for evidence of attack. During each census, we recorded the replica circumstances (as intact, attacked or missing). Whenever an attack was scored, the replica in question was removed without replacement, because we consider that a predation event means that an individual is no longer available to predators. On those occasions when replicas were moved by predators from the initial location, a thorough search was made within a five m radius around the point where the replica was originally placed. Missing replicas were scored as predated and were also not replaced^22,25^. Each attacked replica was inspected with a stereoscopic microscope to reliably identify marks left by predators. Marks made by the potential visually oriented predators (birds, ants, lizards and mammals) are different and distinguishable^11,27,33,90^, A.S.F. personal observation. Birds leave V-shaped marks that lack tooth imprints (Fig. 5a), carnivorous ants leave cut-shaped marks (mandibles, Fig. 5b), lizards leave U-shaped marks with distinct tooth imprints (Fig. 5c), and mammals leave incisor or square-shape tooth marks (Fig. 5d). Cases where marks could not be attributed to a particular class of predator were classified as unknown. In most such cases, the replica had been attacked with such intensity that no specific marks could be used to identify the predator positively. We also recorded the replica body region on which the attack occurred (head, torso, tail or limbs). Replicas with marks in multiple regions of the body were classified as a distinct category (all). Due to the complexity and high richness of predator species in the caatinga, we did not evaluate predators by species, but categorized by predator classes (e.g., birds, lizards and mammals), the exception was the ant *Dinoponera quadriceps*, the only known potential lizard predator ant for the study area.

### Statistical analysis

We used a Principal Component Analysis (PCA) to classify experimental sites according to their structural complexity given the analyzed environmental data by visually checking for overlapping sites in the environmental space. We performed a PCA between the three experimental sites using the eight environmental variables measured for all months to test the general structure. Additionally, we performed a PCA for each season (dry and rainy) to assess the environmental differences caused by seasonality. Because the environmental variables had different scales, we used the command "*scale* = *TRUE*" in the *prcomp* function of the *vegan* R package. We also used a multivariate analysis of variance (MANOVA) to test for significant differences between PCA scores. The first two Principal Components (PCs) of the PCA were used as dependent variables, and experimental sites was used as a factor.

We used generalized linear mixed models (GLMMs) with binomial error distribution to quantify differences in attack rates between habitat complexity and season. We used the attack rates (1—attacked or 0—not attacked) as a binary response variable (dependent variable), habitat complexity (S1, S2 and S3) and seasons (dry and rainy) as explanatory variables (fixed effects), experimental sites (stations) and microhabitats (soil, rock and tree) as random effects. The absolute frequencies of attacks on replica body parts (head, torso and tail) were compared to the expected frequencies calculated from their relative surfaces, by means of a *x*^2^ goodness-of-fit test. In the predator class, we only consider birds, because lizards and mammals had less than five attacks on the replicas (four and three attacks, respectively), and although we recorded 14 attacks by the giant ant (*Dinoponera quadriceps*), ants are not counted as lizard predators^11,33,34^. All statistical analyses were performed with R 3.5.0 software^91^, and we used α < 0.05 to assess significance.

## Supplementary Information

Supplementary Information.

## Data Availability

Data will be made available from the Dryad Digital Repository.
