# Supplementary material for: Predation risk is a function of seasonality rather than habitat complexity in a tropical semiarid forest
Source: Sci Rep. 2021 Aug 17;11:16670. doi: 10.1038/s41598-021-96216-8 (PMC8371019; doi:10.1038/s41598-021-96216-8)
Supplement: Supplementary file 1 — Supplementary Information. [file 41598_2021_96216_MOESM1_ESM.pdf]

Supplementary Information

Article title: Predation risk is a function of seasonality rather than habitat complexity in a tropical semiarid forest

Journal name: Scientific Reports

Author names: Anthony Santana Ferreira[1,2*] and Renato Gomes Faria[3]

Affiliations: [1]Programa de Pós-Graduação em Ecologia e Conservação, Universidade Federal de Sergipe, São Cristóvão-Sergipe, Brazil.

[2]Programa de Capacitação Institucional, Instituto Nacional de Pesquisas da Amazônia (INPA), 69067-375, Manaus, Amazonas, Brazil.

[3]Departamento de Biologia, Universidade Federal de Sergipe (UFS), 49100-000, São Cristóvão, SE, Brazil. Email: renatogfaria@gmail.com

*Corresponding author: anthonyyferreira@gmail.com

Figures

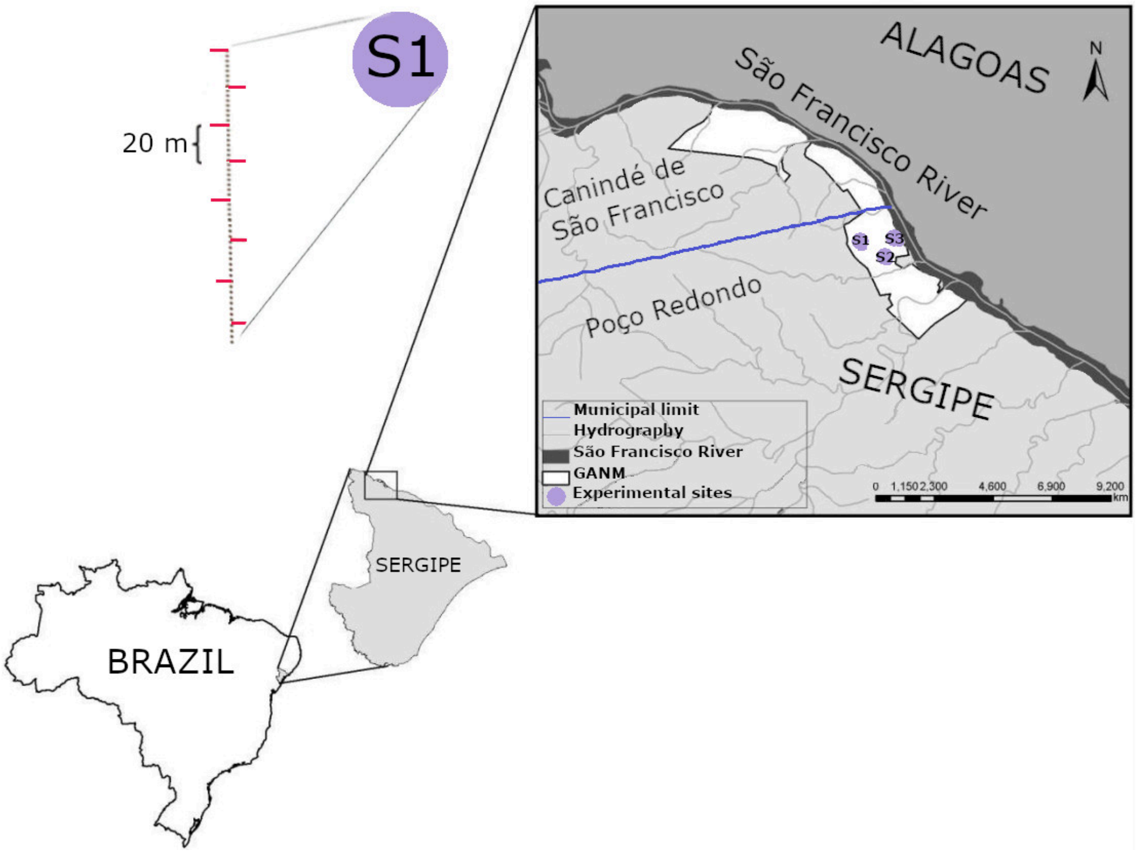

Figure S1 Location of the Grota do Angico Natural Monument - GANM in the state of Sergipe, northeastern Brazil. Violet circles indicate the distribution of the experimental sites (S1, S2, S3) from which eight standardized sampling stations (red lines) were installed along each one. At each station, were positioned 20 lizard replicas (about 3-5 m spacing), a total of 160 replicas per site (480 replicas per season and 960 in total). Map generated using QGIS v2.18 (http://www.qgis.org).

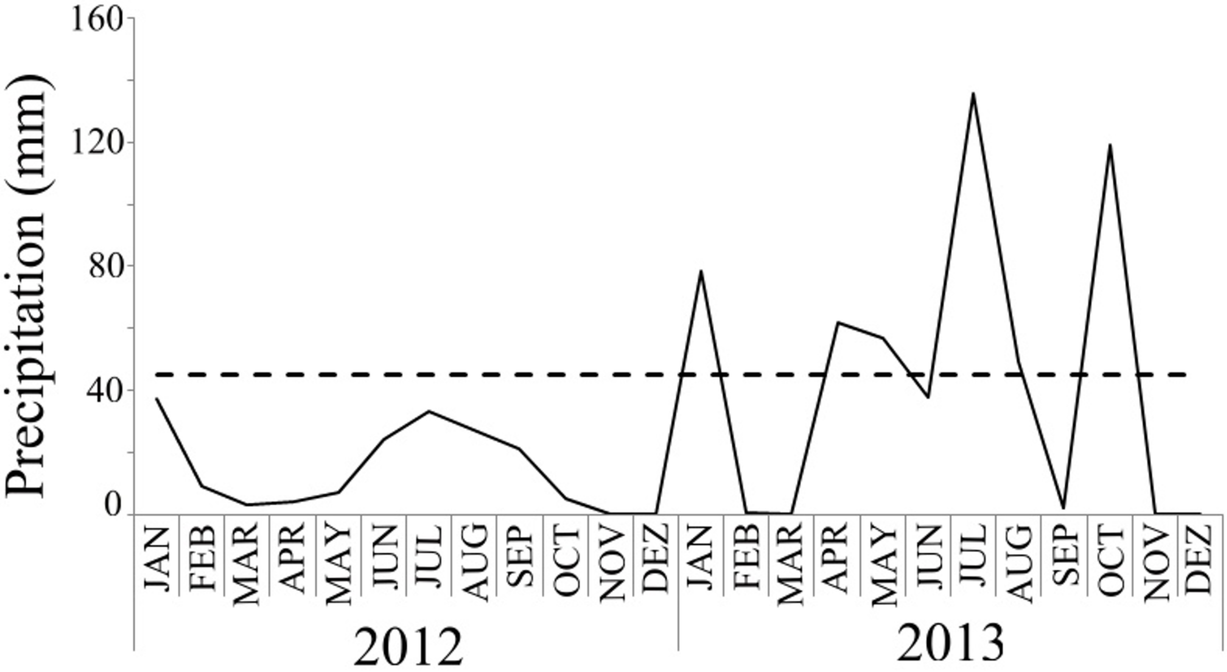

Figure S2 Rainfall data of 2012 and 2013 (source: Secretaria de Meio Ambiente e Recursos Hídricos - SEMARH/SE) from the Poço Redondo rainfall station. Graph generated using Excel v15.26 (https://www.microsoft.com/excel).

Tables

Table S1 Loading resulting from the Principal Component Analysis conducted with eight environmental variables (scaled) throughout all months (general structure) of the three study sites at the Grota do Angico Natural Monument, Sergipe, Brazil. Abbreviation: (PC) principal component. (EXS) exposed soil. (LEL) leaf litter. (NUS) number of stems. (DNT) distance of nearest tree. (DBH) diameter at breast height of nearest tree. (NFT) number of fallen trunks. (BRO) bromeliads.

	PC1	PC2	PC3	PC4	PC5	PC6	PC7	PC8
EXS	-0.3056	0.4809	-0.3251	0.0466	-0.0721	0.3698	0.3979	-0.5170
LEL	0.1637	-0.4624	0.5532	-0.0901	-0.2555	0.4112	0.3756	-0.2641
ROCK	0.3688	-0.3520	-0.3286	0.1922	0.4879	-0.3190	0.3982	-0.3112
NUS	0.4515	0.2020	-0.0938	-0.2809	-0.6190	-0.4499	-0.0073	-0.2864
DNT	-0.4630	-0.2340	-0.1042	0.3162	-0.4285	-0.3592	0.4612	0.3077
DBH	-0.3850	-0.4439	-0.1307	0.1148	-0.1315	-0.0879	-0.5411	-0.5538
NFT	0.2749	0.2522	0.2580	0.8640	-0.1159	0.0214	-0.1546	-0.0998
BRO	0.3190	-0.2736	-0.6140	0.1191	-0.3066	0.5028	-0.1122	0.2698
Variance (%)	36.3	28	13.7	9.3	6.3	2.7	2.1	1.6
Cumulative (%)	36.3	64.3	78	87.3	93.6	96.3	98.4	100

Table S2 Loading resulting from the Principal Component Analysis conducted with eight environmental variables (scaled) throughout dry season (10 months) of the three study sites at the Grota do Angico Natural Monument, Sergipe, Brazil. Abbreviation: (PC) principal component. (EXS) exposed soil. (LEL) leaf litter. (NUS) number of stems. (DNT) distance of nearest tree. (DBH) diameter at breast height of nearest tree. (NFT) number of fallen trunks. (BRO) bromeliads.

	PC1	PC2	PC3	PC4	PC5	PC6	PC7	PC8
EXS	-0.3727	-0.4266	-0.3487	0.0149	-0.0586	0.1011	-0.0768	0.7333
LEL	0.3046	0.4310	0.4758	-0.1511	0.0419	0.0014	0.1928	0.6583
ROCK	0.4287	0.1791	-0.3335	0.5089	0.0479	-0.4313	-0.4436	0.1699
NUS	0.4168	-0.2211	-0.1039	-0.6614	0.2749	0.1474	-0.4815	-0.0016
DNT	-0.3941	0.2531	-0.0792	-0.0233	0.8601	-0.1825	0.0269	0.0064
DBH	-0.2491	0.5418	-0.0961	0.0988	-0.1321	0.6276	-0.4627	-0.0047
NFT	0.1968	-0.4259	0.4245	0.5165	0.3644	0.4375	-0.0932	0.0027
BRO	0.3933	0.1110	-0.5782	0.0580	0.1644	0.4043	0.5522	0.0035
Variance (%)	39.5	28.5	14.6	7.1	5.9	2.4	2	0
Cumulative (%)	39.5	68.0	82.5	89.7	95.6	98	100	100

Table S3 Loading resulting from the Principal Component Analysis conducted with eight environmental variables (scaled) throughout rainy season (eight months) of the three study sites at the Grota do Angico Natural Monument, Sergipe, Brazil. Abbreviation: (PC) principal component, (EXS) exposed soil, (LEL) leaf litter, (NUS) number of stems, (DNT) distance of nearest tree, (DBH) diameter at breast height of nearest tree, (NFT) number of fallen trunks, (BRO) bromeliads.

	PC1	PC2	PC3	PC4	PC5	PC6	PC7	PC8
EXS	-0.1541	-0.5023	0.1550	-0.5369	-0.5833	0.1180	-0.2158	-0.1048
LEL	-0.1296	0.3411	-0.7615	-0.2497	-0.1092	0.4513	-0.0444	-0.0841
ROCK	0.1821	0.5421	0.2308	0.2537	-0.4882	0.0096	-0.5601	0.0567
NUS	0.4701	-0.1772	0.0908	-0.2142	0.5224	0.3769	-0.5278	-0.0064
DNT	-0.5214	0.1242	0.0603	-0.2530	0.2250	-0.1205	-0.2650	0.7140
DBH	-0.4749	0.2629	0.2026	-0.1835	0.2931	-0.1730	-0.2271	-0.6806
NFT	0.4189	0.1439	-0.2751	-0.4625	-0.0075	-0.7151	-0.0529	0.0022
BRO	0.1801	0.4482	0.4629	-0.4770	-0.0055	0.2906	0.4844	0.0760
Variance (%)	39.5	28	12.3	9.1	4.2	4.1	2.4	0.5
Cumulative (%)	39.5	67.4	79.7	88.8	93	97.1	99.5	100

Table S4 Number of lizard replicas used in each experimental site with the overall attacks and in both seasons (upper panel); number of attacks on different body parts of the replicas (middle panel) and number of attacks on replicas placed on different substrates (lower panel). The rows in italics are to highlight the fact that they are subrows of the upper row.

Models	S1 overall	S2 overall	S3 overall	Total overall	S1 rainy	S2 rainy	S3 rainy	Total rainy	S1 dry	S2 dry	S3 dry	Total dry	
Not attacked	273	269	268	810	145	141	151	437	128	128	117	373	
Attacked	44	47	39	130	14	17	6	37	30	30	33	93	
by birds	24	34	36	94	10	16	5	31	14	18	31	63	
by ants	9	5	0	14	2	0	0	2	7	5	0	12	
by mammal	2	0	1	3	0	0	0	0	2	0	1	3	
by lizards	3	1	0	4	0	0	0	0	3	1	0	4	
undetermined	6	7	2	15	2	1	1	4	4	6	1	11	
Missing	3	4	13	20	1	2	3	6	2	2	10	14	
Total	320	320	320	960	160	160	160	480	160	160	160	480	
Model parts attacked	S1 overall	S2 overall	S3 overall	Total overall	S1 rainy	S2 rainy	S3 rainy	Total rainy	S1 dry	S2 dry	S3 dry	Total dry	Expected attacks
Head	37	41	36	114	12	14	6	32	25	27	30	82	225.04
Torso	27	23	16	66	9	11	1	21	18	12	15	45	438.44
Tail	6	5	3	14	1	2	0	3	5	3	3	11	791.52
Total	70	69	55	194	22	27	7	56	48	42	48	138	
Substrates	S1 overall	S2 overall	S3 overall	Total overall	S1 rainy	S2 rainy	S3 rainy	Total rainy	S1 dry	S2 dry	S3 dry	Total dry	Total placed
Soil	24	25	21	70	5	3	1	9	19	22	20	61	634
Rock	3	2	8	13	1	2	1	4	2	0	7	9	226
Tree	17	20	10	47	8	12	4	24	9	8	6	23	100
Total	44	47	39	130	14	17	6	37	30	30	33	93	960

Table S5 Result of the models that examine whether the differences between habitat structural complexity and seasonality explained the differences in predation rates of lizard replicas taking into account predations by the giant ant *Dinoponera quadriceps*.

Between experimental sites: intercept ± SE = -1.99 ± 0.58, Wald test, $P < 0.001$; experimental sites (S1) ± SE = -0.09 ± 0.23, Wald test, $P = 0.72$; (S3) ± SE = -0.21 ± 0.24, Wald test, $P = 0.39$
Between seasons: intercept ± SE = −1.29 ± 0.11, Wald test, $P < 0.001$; seasonality ± SE = -1.59 ± 0.23, Wald test, $P < 0.001$
